# Human osteocyte expression of Nerve Growth Factor: The effect of Pentosan Polysulphate Sodium (PPS) and implications for pain associated with knee osteoarthritis

**DOI:** 10.1371/journal.pone.0222602

**Published:** 2019-09-26

**Authors:** Catherine J. M. Stapledon, Helen Tsangari, Lucian B. Solomon, David G. Campbell, Plinio Hurtado, Ravi Krishnan, Gerald J. Atkins

**Affiliations:** 1 Centre for Orthopaedic & Trauma Research, The University of Adelaide, Adelaide, South Australia, Australia; 2 Orthopaedic and Trauma Service, Royal Adelaide Hospital, Adelaide, South Australia, Australia; 3 Wakefield Orthopaedic Clinic, Calvary Wakefield Hospital, Adelaide, South Australia, Australia; 4 Renal Unit, Royal Adelaide Hospital, Adelaide, South Australia, Australia; 5 Paradigm Biopharmaceuticals Ltd., Melbourne, Victoria, Australia; Universite de Nantes, FRANCE

## Abstract

Pentosan polysulphate sodium (PPS) is a promising therapeutic agent for blocking knee pain in individuals with knee osteoarthritis (KOA). The mode of action of PPS in this context is unknown. We hypothesised that the osteocyte, being the principal cell type in the sub-chondral bone, was capable of expressing the pain mediator Nerve Growth Factor (NGF), and that this may be altered in the presence of PPS. We tested the expression of NGF and the response to PPS in the presence or absence of the proinflammatory cytokine tumour necrosis factor-alpha (TNFα), in human osteocytes. For this we differentiated human primary osteoblasts grown from subchondral bone obtained at primary knee arthroplasty for KOA to an osteocyte-like stage over 28d. We also tested NGF expression in fresh osteocytes obtained by sequential digestion from KOA bone and by immunofluorescence in KOA bone sections. We demonstrate for the first time the production and secretion of NGF/proNGF by this cell type derived from patients with KOA, implicating osteocytes in the pain response in this pathological condition and possibly others. PPS inhibited TNFα-induced levels of proNGF secretion and TNFα induced *NGF* mRNA expression. Together, this provides evidence that PPS may act to suppress the release of NGF in the subchondral bone to ameliorate pain associated with knee osteoarthritis.

## Introduction

Osteoarthritis of the knee (KOA) is a common and painful condition, for which the first line of management is the prescription of analgesics to control pain. The aetiology of KOA is incompletely understood but is known to be associated with the increased expression of proinflammatory mediators, including tumour necrosis factor alpha (TNFα) and interleukin 1-beta (IL-1β) [1]. These are thought to stimulate the localised production of cartilage-degrading enzymes, such as matrix metallopeptidase (MMP) family member-13 (MMP-13). After exhaustion of nonoperative management options, KOA is ultimately treated by performing a total knee arthroplasty (TKA), in which the diseased joint is replaced by a prosthesis. Patient reported pain is the major indicator for TKA [2].

Bone is a well innervated tissue, with bone sensory neurons deriving solely from the dorsal root ganglia of the spinal cord [3]. The secreted neurotropic protein beta-Nerve Growth Factor (NGF) is a major contributor to pain in a number of chronic conditions, including KOA [4–6]. Furthermore, NGF mRNA expression is known to be induced by both TNFα and IL-1β in an experimental mouse model of osteoarthritis [7]. NGF binds to at least two receptors expressed by neurons, tropomyosin receptor kinase A (TrkA) and the pan-neurotropin receptor p75/NTR, where it can have diverse biological effects, either promoting neuronal growth or causing neuron apoptosis, depending on whether the neuron also expresses the co-receptor, sortilin [8]. A neutralising antibody treatment that sequesters NGF, tanezumab®, has yielded promising results in the treatment of pain associated with KOA [1, 4–6], consistent with NGF being both a key readout and a mediator of pain for this condition. NGF is first translated as a pro-protein form (proNGF), which is post-translationally processed (proteolytically cleaved) to the mature form by the action of furin or furin-like pro-protein convertases [8]. Currently, the accurate detection of soluble NGF levels using commercially available enzyme linked immunosorbent assays (ELISAs) is problematic due to the influence on readouts of proNGF; if present, proNGF interferes with the readouts of a number of commercially available ELISA kits in an unpredictable fashion, in terms of both the magnitude and the direction (increase or decrease) of the effect [9]. It is therefore necessary to defer quantitative assessment to the levels of proNGF [9].

Pentosan Polysulphate Sodium (PPS) is an FDA-approved drug for the treatment of interstitial cystitis and bladder pain syndrome, with an excellent safety profile [10]. It is currently being tested for its efficacy as a treatment for KOA with promising results [11, 12]. The mode of action of PPS appears to be multi-factorial, and includes replenishment of the glycosaminoglycan (GAG) layer in the case of its effect in interstitial cystitis, as well as effects on intracellular signalling, in particular the nuclear factor kappa-B (NFκB) [13] and the IL-1β-iNOS [14] pathways in chondrocytes. Importantly, KOA is a disease of the entire joint, with changes to the sub-chondral bone, as well as to the synovium and cartilage [1]. The contribution of each tissue to disease progression and to the associated pain is incompletely understood. In advanced KOA, there is nearly complete degradation of the cartilage with a paucity of healthy chondrocytes remaining. This suggests that mediators of pain may derive to a significant extent from the underlying sub-chondral bone. The most numerous cell type in hard bone tissue is the osteocyte, and these cells are increasingly recognised as the key controlling cell type in many local and systemic physiological processes [15, 16]. In conditions associated with osteoarthritis, the osteocyte is involved in the inflammatory, osteolytic response to implant-derived wear particles [17] and also elicits impressive pro-inflammatory responses to bacteria in the condition of periprosthetic joint infection [18].

We hypothesised that osteocytes are capable of producing NGF in the inflammatory milieu of the subchondral bone in KOA and that PPS may act by inhibiting this production. To test this hypothesis, we examined the expression of NGF in freshly isolated human primary osteocytes. We then tested the effects of PPS on the responses of human primary osteocyte-like cultures, differentiated from the proximal tibiae of patients suffering from advanced KOA and undergoing TKA. Treatment with recombinant TNFα was used as the proinflammatory stimulus, and the effects on the relative expression of *NGF* mRNA was examined. ProNGF protein levels were also determined. We show for the first time that human osteocytes are capable of producing NGF, suggesting that they potentially contribute to localised pain responses. We also show that PPS suppresses *NGF* mRNA transcription and proNGF secretion by osteocytes and reverses the stimulatory effects of TNFα on these processes. Together, our findings suggest a hitherto unknown role for osteocytes in the pain response and a mechanism for the pain benefit in KOA patients taking PPS.

## Materials and methods

### Ethical statement

All studies with human patient derived material were covered by pre-existing ethics committee approval by the Human Research Ethics Committees of the Royal Adelaide Hospital (Approval No. 130114) and Calvary Health Care Limited (Approval No. 13-CHREC-E006). All donor material was obtained with written informed patient consent.

### Donors and osteocyte-like cells

In order to represent the clinical relevance of the findings from this study, the effects of PPS were to be tested on osteocyte-like cultures [17, 19–23] derived by differentiation *in vitro* for a period of 28 days from cells isolated from the subchondral bone of the proximal tibia of three patients with advanced knee OA who underwent total knee arthroplasty (TKA) surgery (KOA). To examine potential differences with non-OA bone, cells were also isolated from the proximal femur of three patients who underwent total hip arthroplasty (THA) for neck of femur fracture (NOF). The gender of all donors was female, and groups were age-matched, with the mean age of KOA being 77.0 ± 8.5 years and that of the NOF group being 77.7 ± 5.5 years (*p* = 0.91).

Cryopreserved cells from each donor (all at passage 0 or 1) were thawed and cultured for 10 days in T75 cm^2^ tissue culture flasks. Once confluent, cells were removed by collagenase/dispase digestion, washed by centrifugation, counted and adjusted to 5 x 10^5^ cells/ml. Cells were then seeded into either 12-well tissue culture trays or into 8-well chamber slides, at 1 x 10^5^ and 2 x 10^4^ cells/well, respectively. After 24h, media were replaced with osteogenic differentiation medium, consisting of αMEM, 5% v/v foetal calf serum (FCS), 1.8 mM potassium dihydrogen phosphate (KH_2_PO_4_), 100 μM Ascorbate-2-phosphate (As2P), 10 mM HEPES, 1 x 10^−8^ M Dexamethasone and 0.2 mM L-Glutamine. During the differentiation process, samples were collected at days 3, 14 and 28 in Trizol reagent for total RNA preparation and gene expression analysis (see below). Cultures seeded into chamber slides were examined for *in vitro* mineralisation using the Alizarin Red staining technique, as previously described [22].

### PPS and TNFα treatments

PPS (bene pharmaChem GmbH & Co. KG, Geretstried, Germany) was dissolved in sterile PBS as a stock solution at 1.0 mg/ml. Differentiated cells were either untreated or pre-treated with final concentrations of PPS at 1, 5 or 50 μg/ml in culture medium for 72h. The tested doses of PPS were based on the effective and maximally active levels published in a previous study [24]. Media were then replaced with the same concentrations of PPS with or without the addition of recombinant human (rh) TNFα (1 ng/ml) and then cultured for a further 48h. Culture supernatants were collected and total RNA and cDNA prepared, as described below.

### Isolation of human osteocytes

Osteocytes were isolated directly from human KOA bone samples (*n* = 4), according to our published protocol [25]. Briefly, bone obtained from TKA was rinsed vigorously in sterile PBS and then subjected to six serial digestions of collagenase/dispase/EDTA, with intervening recovery of released cells by centrifugation and washing in PBS. The cells obtained from digests 4–6, corresponding to an osteocyte-enriched fraction [25] were pooled, washed twice further by centrifugation and resuspension in PBS and then seeded into 8-well glass bottomed chamber slides. After allowing cells to recover for 72h, they were either immunostained or pretreated with PPS and then treated with combinations of PPS and rhTNFα, as indicated.

### Gene expression analysis

Total RNA was prepared from Trizol lysates, according to the manufacturer’s instructions, with the exception that due to evidence for PPS interference in the generation of assayable cDNA, RNA precipitates were washed 3 times in 75% ethanol instead of the usual single wash step, in an attempt to remove residual PPS. RNA preparations were tested for yield and purity using a Nanodrop microvolume spectrophotometer (Thermo Fisher). One microgram of RNA from each sample was reverse transcribed using a Superscript™ II kit (Thermo Fisher), as per manufacturer’s instructions. Real-time RT-PCR was performed for genes including Nerve Growth Factor (*NGF*), its receptors *NTRK1* (*TRKA*) and *NGFR* (*P75NTR*), *MMP13*, *RANKL*, *OPG*, *OCN*, *DMP1* and *SOST*, relative to housekeeping gene (*ACTB*) expression. Oligonucleotide primer sequences for each of these are shown in **Table 1**.

**Table 1:** Human mRNA-specific oligonucleotide primer sequences.

Gene	Forward Primer Sequence	Reverse Primer Sequence
*ACTB* [17]^a^	5′‐cgcgagaagatgacccagatc‐3′	5′‐tcaccggagtccatcacg‐3′
*DMP1* [17]	5′‐gatcagcatcctgctcatgtt‐3′	5′‐agccaaatgacccttccattc‐3′
*MMP13* [17]	5′‐ggatccagtctctctatggt‐3′	5′‐ggcatcaagggataaggaag‐3′
*NGF* **[26]**	5′‐cacactgaggtgcatagcgt‐3′	5′‐tgatgaccgcttgctcctgt‐3′
*NGFR/TRKA* **[27]**	5′‐cctggacagcgtgacgttc‐3′	5′‐cccagtcgtctcatcctggt‐3′
*P75NTR* **[27]**	5′‐cctggacagcgtgacgttc‐3′	5′‐cccagtcgtctcatcctggt‐3′
*OCN* [17]	5′‐atgagagccctcacactcctcg‐3′	5′‐gtcagccaactcgtcacagtcc‐3′
*OPG* [17]	5′‐gctcacaagaacagactttccag‐3′	5′‐ctgttttcacagaggtcaatatctt‐3′
*RANKL* [17]	5′‐ccaagatctccaacatgact‐3′	5′‐tacaccattagttgaagatact‐3′
*SOST* [17]	5′‐accggagctggagaacaaca‐3′	5′‐gctgtactcggacacgtctt‐3′

^a^Published references to primer pairs are indicated next to gene names; all primer pairs were designed and/or validated in-house.

### ELISA analysis

Culture supernatants were stored frozen (-80°C) until use, whereupon they were thawed at 4°C, and assayed by ELISA for human NGF (Cat. No: EHNGF; Thermo Fisher Scientific) or proNGF (Cat. No: BEK-2226-2P; Biosensis) protein levels, as per the manufacturers’ instructions.

### Immunostaining

Cells seeded in 8-well chamber slides and differentiated for 28 days or freshly digested from bone were either untreated or treated with rhTNFα (1 ng/ml), PPS (50μg/ml; ‘PPS50’) or PPS50 + rhTNFα, as indicated. For immunostaining, media were removed and wells rinsed three times with PBS (pH 7.4). Cells were then fixed with 100μl of Histochoice (Sigma-Aldrich) tissue fixative for 1 hour at room temperature. After fixation, wells were rinsed twice with distilled H_2_O and stored at 4°C until staining. Cells were blocked with 50μl of blocking buffer (5% v/v normal rabbit serum in 1 x PBS) for 20 minutes at room temperature in a humid chamber. Cells were then rinsed with wash buffer (0.1% v/v normal rabbit serum in PBS) three times. Cells were stained with either mouse monoclonal antibody (MAb) *anti*-human NGF (25623; Thermo Fisher Scientific), *anti*-human NGFR (2F1C2; Thermo Fisher Scientific), *anti*-human TrkA (6B2; Thermo Fisher Scientific), or *anti*-human SOST (MAb 220902.11; R&D Systems, Minneapolis, MN, USA) primary antibodies and their respective isotype controls (IgG_1_; MAb 1B5), diluted as indicated. Cells were incubated with primary antibody for 40 minutes at 4°C. For unconjugated MAbs, chamber slides were then rinsed 3 x with wash buffer and 50μl of rabbit α-mouse Alexa-fluor secondary antibody (1:2000 dilution), also containing nuclear DAPI stain (1:2000 dilution; diamidino-2-phenylindole; Thermo Fisher) was added for 1h at room temperature. Wells were then washed three times with wash buffer.

Finally, FluoroBrite DMEM (Life Technologies) was added to each well to image using confocal microscopy (FV3000 Confocal Microscope, Olympus Lifescience).

For double-labelling purposes, anti-NGF was directly conjugated to fluorescein isothiocyanate (FITC; Sigma Chemical Co., St. Louis, MO, USA). For this MAb 25623 was first dialysed against carbonate/bicarbonate buffer (1l; pH 9.6) at 4°C overnight. FITC was dissolved to 1mg/ml in anhydrous DMSO. 15μl FITC solution was added to 100μg anti-NGF and the tube mixed on a rotator for 2 h at room temperature. Unbound FITC was removed using size-exclusion chromatography on a Sephadex G-25 column (Pharmacia Biotech, Piscataway, NJ, USA). The absorbance of 0.5 ml fractions at 280nm and 492nm was determined using a NanoDrop One spectrophotometer (Thermo Fisher Scientific) and the concentration of FITC-conjugated antibody determined by the formula: concentration (mg/ml) = A_280_ –(A_492_ x 0.35)/1.4. To remove aggregates, the antibody solution was centrifuged at 16,400 RCF for 15 minutes prior to use. As a positive control for immunostaining, we identified the small cell lung carcinoma cell line NCI-H266 (ATCC, Masassas, VA, USA) to be NGF-expressing using the Harmonizome database [28]. For these assays, FITC-conjugated X-63 MAb (Biosensis, Thebarton, SA, Australia) was used as a negative control; direct conjugates were incubated for 40 min, aspirated and the wells washed three times, as above.

Bone isolated from KOA patients was fixed, decalcified, embedded and sectioned, as described [18]. Bone sections (5 μm) were first heated at 60°C for 15 min to melt excess paraffin and then dewaxed. For antigen retrieval, slides were then incubated in 10% formic acid in distilled water for 10 min, rinsed in PBS and then immunostained, as above.

### Data and statistical analysis

Data were analysed by two-way analysis of variance (ANOVA) with Holm-Sidak’s multiple comparison post-hoc tests using GraphPad Prism software (GraphPad Prism, La Jolla, CA, USA). Values for *p* < 0.05 were considered statistically significant.

## Results and discussion

### NGF expression by cultured osteocytes

**Fig 1:**
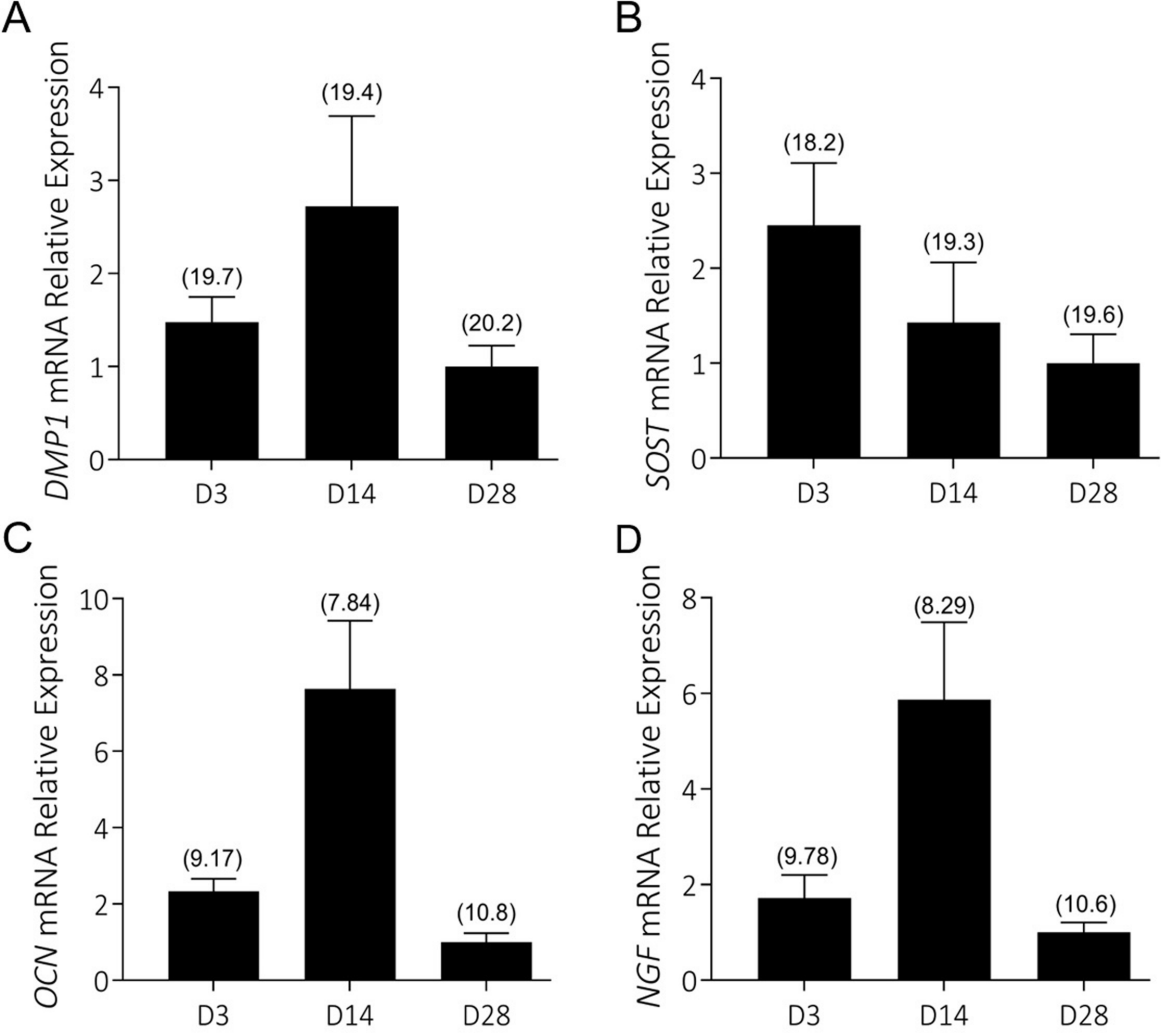
Human differentiating KOA osteoblast/osteocyte cultures express NGF: Cells from 3 KOA patients were cultured under pro-osteogenic conditions for up to 28d, as described in Materials and Methods. Gene expression was measured by real-time RT-PCR at various timepoints for: A) *DMP1*; B) *SOST*; C) *OCN*; D) *NGF*. Data (means + standard error of the mean (SEM)) were normalised to the expression of *18S* rRNA using the 2^-(ΔCT)^ method and are shown relative to the expression of each gene at the end of the time course pooled from 3 donors’ cells. The mean ΔCT for each timepoint is indicated in parentheses above each histogram.

Human primary osteoblasts isolated from the subchondral bone of patients undergoing TKA for osteoarthritis of the knee (KOA) or THA for neck of femur fracture (NOF) were cultured under differentiating conditions for a period of 28d [16–21, 29]. Overall the NOF cultures mineralised to a significantly greater extent than KOA donors, as assayed by Alizarin Red staining (**S1 Fig**). The reason for this could relate either to the site harvested (proximal femur for NOF and subchondral proximal tibia for KOA), or more likely, to the dysregulated mineralisation evident in differentiating osteoblasts from patients with osteoarthritis, as we have previously reported for cells isolated from the proximal femur [22]. All KOA donors’ cells displayed strong characteristics of pre-osteocytes/osteocytes by day 3, expressing appreciable mRNA for *DMP1*, *SOST* and *OCN* (**Fig 1A–1C**). *DMP1* and *SOST* mRNAs were expressed to a similar level overall based on delta-cycle threshold (ΔCT) values, while *OCN* mRNA was more abundantly expressed. The expression of *DMP1* and *OCN* increased by D14 and then declined by D28, consistent with the acquisition of a mature osteocyte-like phenotype associated with loss of organelles and a decrease in the overall metabolic level [16, 30]. The expression of *NGF* mRNA was relatively abundant in these cultures, similar to that of *OCN*, from all donors’ cells tested and mirrored that of the differentiation markers above, peaking at day 14 and declining by day 28 (**Fig 1D**). Furthermore, all KOA donors’ cells secreted appreciable full-length NGF protein detected in the supernatant (111.9 ± 48.8 pg/ml).

### NGF expression by freshly isolated human osteocytes and in human bone

**Fig 2:**
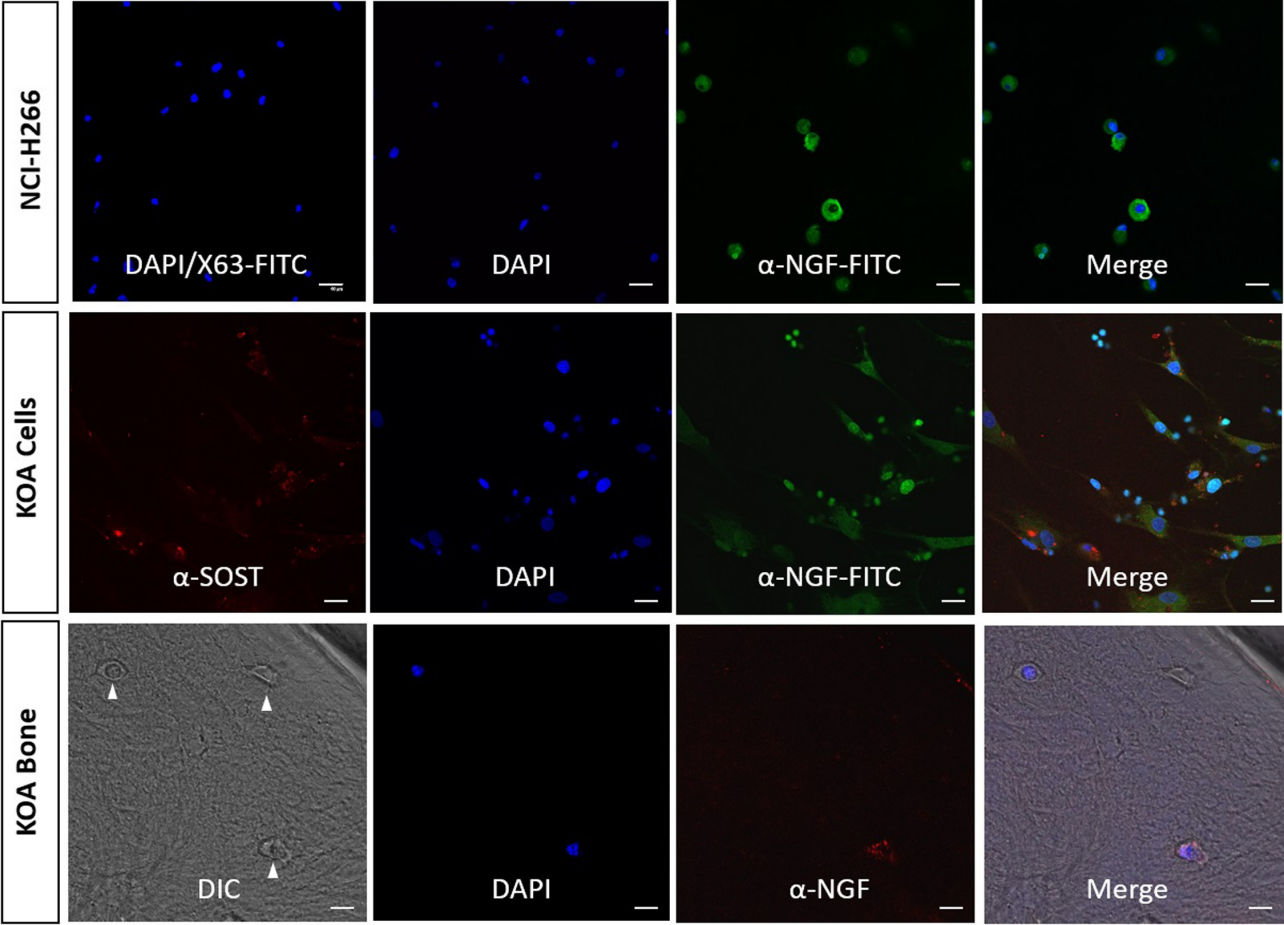
NGF expression in isolated human osteocytes and in KOA bone. Directly conjugated α-NGF MAb was tested against NCI-H266 cells and compared against a directly conjugated negative control antibody, X-63 (*upper row*). Fractions IV-VI of a KOA bone digest were similarly stained for NGF, and staining compared against the expression of the osteocyte marker SOST/sclerostin (*middle row*). Dual staining revealed intracellular but not co-localised staining for both NGF and SOST in these cells. Specificity of staining was confirmed using negative control IgG_1_ MAb. Finally, NGF positivity was evident in osteocytes (white arrows) *in situ* in decalcified KOA bone (*bottom row*), here using unconjugated α-NGF MAb, as described in Materials and methods. Bone morphology is revealed by digitial interference contrast (DIC). In all cases nuclei were visualised by DAPI stain (blue). Scale bars represent 50 μm.

To examine NGF protein expression, we optimised staining of a directly conjugated anti-NGF antibody to NCI-H266 cells (**Fig 2**, *top row*). NGF expression was also tested in cells obtained by sequential digestion from human KOA bone. We have published previously that fractions IV-VI obtained using this method are enriched for mature osteocytes [25]. As shown in Fig 2. (*middle row*), numerous cells in the osteocyte-enriched fractions stained brightly for NGF expression and co-stained for the osteocyte marker SOST/sclerostin. Furthermore, osteocyte expression of NGF was evident in stained sections of human KOA subchondral bone (**Fig 2**, *bottom row*). Together with the observations in differentiated cultures above, this is the first report to our knowledge of NGF expression by human osteocytes. A previous study using a fluorescence reporter system to identify NGF expression in mouse bone, reported osteoblast but not osteocyte expression of NGF in response to mechanical loading of the ulna [3]. It is possible that the reporter system used lacked the sensitivity to detect low levels of NGF, or that the difference observed is due to interspecies, relative age, skeletal site, stimulus (mechanical rather than pro-inflammatory), as well as the influence of osteoarthritis on osteocyte expression.

**Fig 3:**
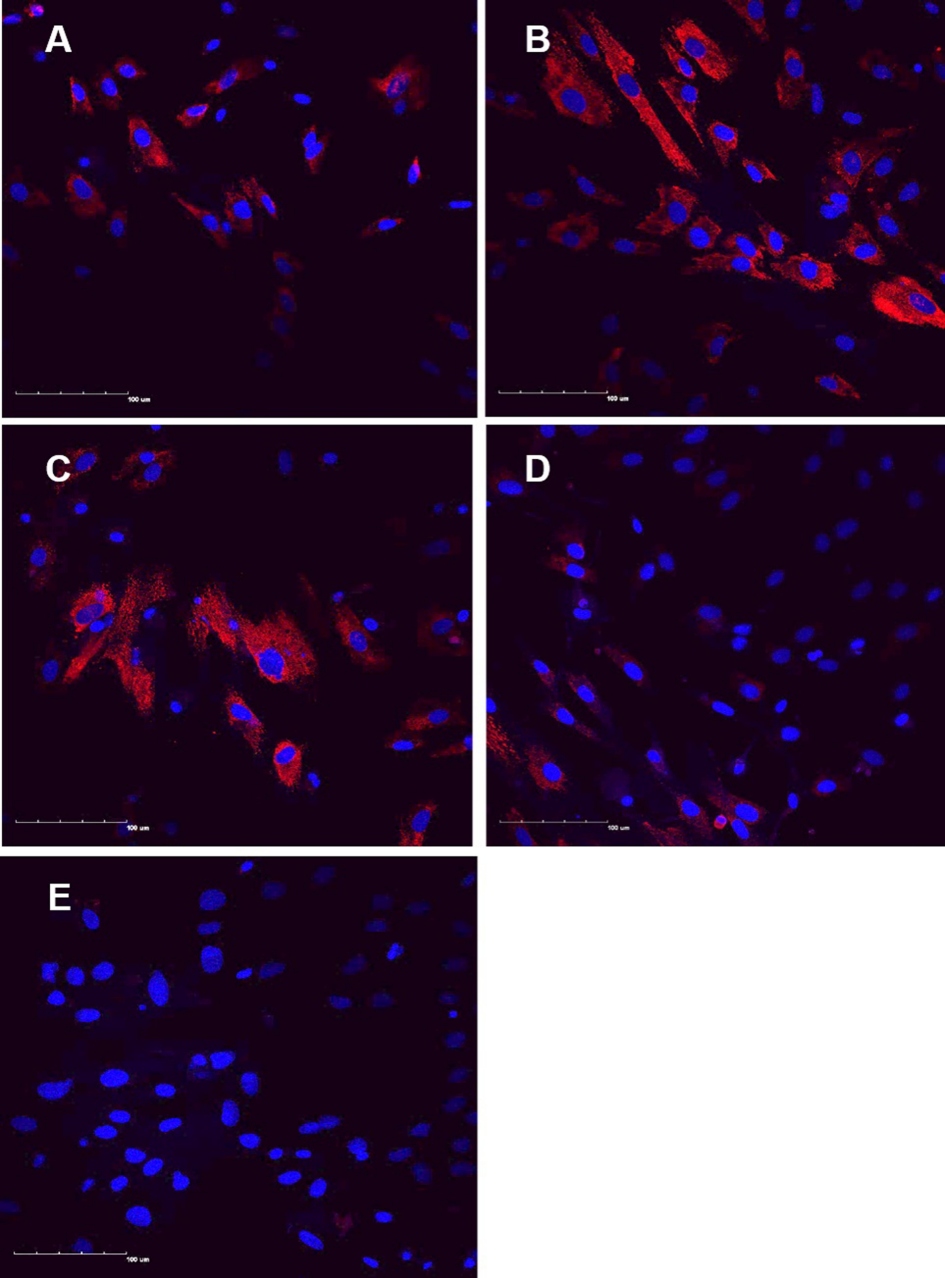
NGF expression in isolated human osteocytes. Osteocyte-enriched fractions of sequential human trabecular bone digests were cultured for 24h either untreated (A), treated with rhTNFα (B), PPS (0.5μg/ml) (C) or a combination of both (D), and then examined by confocal microscopy for NGF immunoreactivity. Control cells were also immunostained using an isotype-matched negative control primary antibody (E). Images are representative of data obtained for four individual donors cells. Scale bars in each image represent 100 μm.

In an attempt to examine regulation of NGF protein expression in freshly isolated KOA osteocyte-like cells, they were treated with either rhTNF-α, PPS or a combination of these. NGF immunostaining was detected in all cases although cells exposed to rhTNF-α alone and PPS alone had qualitatively greater NGF expression than control, and cells treated with a combination of rhTNF-α and PPS showed qualitatively less staining (**Fig 3**).

Due to the unpredictable yields of osteocytes from individual patients’ bone, it is technically difficult to achieve identical and sufficient numbers of resulting adherent viable cells between wells for quantitative assessment of treatments. Furthermore, since NGF is a secreted protein, it is difficult to interpret intracellular levels. We therefore studied the quantitative regulation of NGF secretion into the supernatants of differentiated cultures of osteocyte-like cells.

### Effect of PPS on TNF induced proNGF secretion

**Fig 4:**
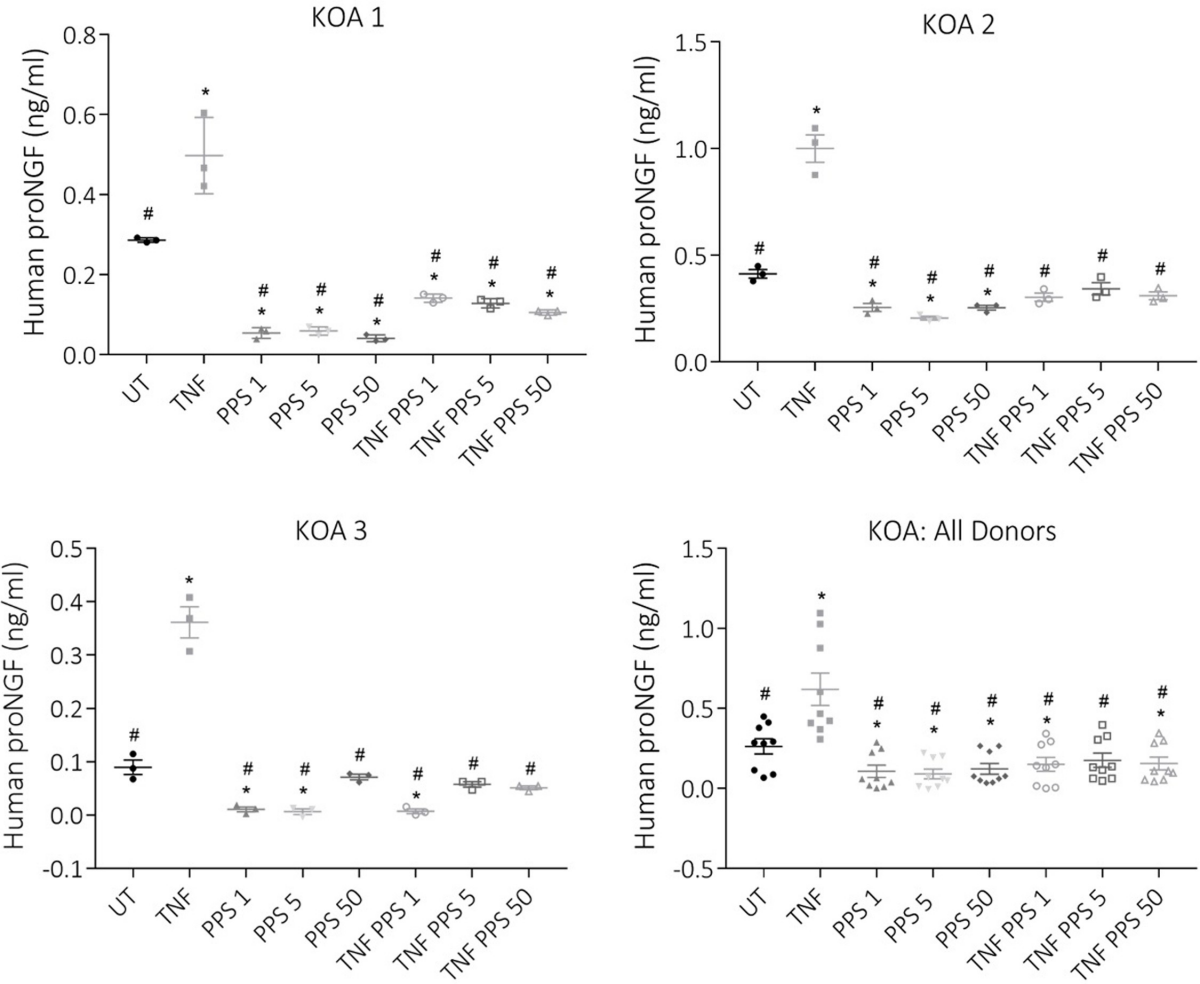
Human osteocyte-like cells secrete proNGF. Secretion of proNGF was tested from cultures of KOA osteocyte-like cells treated with combinations of rhTNF and PPS. Data are means + SD of supernatants harvested from triplicate wells. Significant difference to untreated control (UT) is indicated by *(*p* < 0.05); significant difference to rhTNF treated cultures is indicated by #(*p* < 0.05).

To examine further the effects of PPS on NGF expression, osteocyte-like cultures derived from three KOA donors were treated after 3 days pre-treatment with differing concentrations of PPS, with rhTNFα in the absence or presence of the pre-treatment concentration of PPS. Supernatants were collected 48h following treatment and subjected to ELISA analysis, as described in Materials and Methods. A study by Malerba and colleagues [9] demonstrated that the presence of the immature pro-protein form of NGF, proNGF, together with mature NGF in an experimental sample, imparted false readings in many commercially-available ELISAs for NGF, and these effects were to an unpredictable magnitude and direction. Therefore, in this study proNGF levels were measured in the treated supernatants, as described in Materials and Methods. As was observed for mature NGF protein, basal proNGF was detectable in all donor cell culture supernatants (**Fig 4**). Recombinant human TNFα treatment significantly increased the levels of proNGF, consistent with the induction of NGF expression in response to this pro-inflammatory stimulus in an osteoarthritic setting [7, 31]. PPS strongly suppressed basal proNGF secretion in all donors’ cells assayed at all of the PPS concentrations tested, down to 1 μg/ml. Important from a therapeutic viewpoint, PPS also strongly reversed the effect of rhTNF on proNGF secretion (**Fig 4**), again at all concentrations tested. The concentrations of PPS chosen were based on those reported previously [24]. A study by Dawes *et al*. [32] reported plasma concentrations of PPS of approximately 1–3 μg/ml in volunteers injected subcutaneously with PPS, supportive that the effective doses used here have clinical relevance. However, the lack of a dose response in our assays can be considered a study limitation. Strikingly similar findings were made for NOF cells (**S2 Fig**), suggesting that NGF expression and its regulation by PPS may be a common feature of osteocytes between skeletal sites and pathologies.

### Effect of PPS on TNFα-induced *NGF* mRNA expression

**Fig 5:**
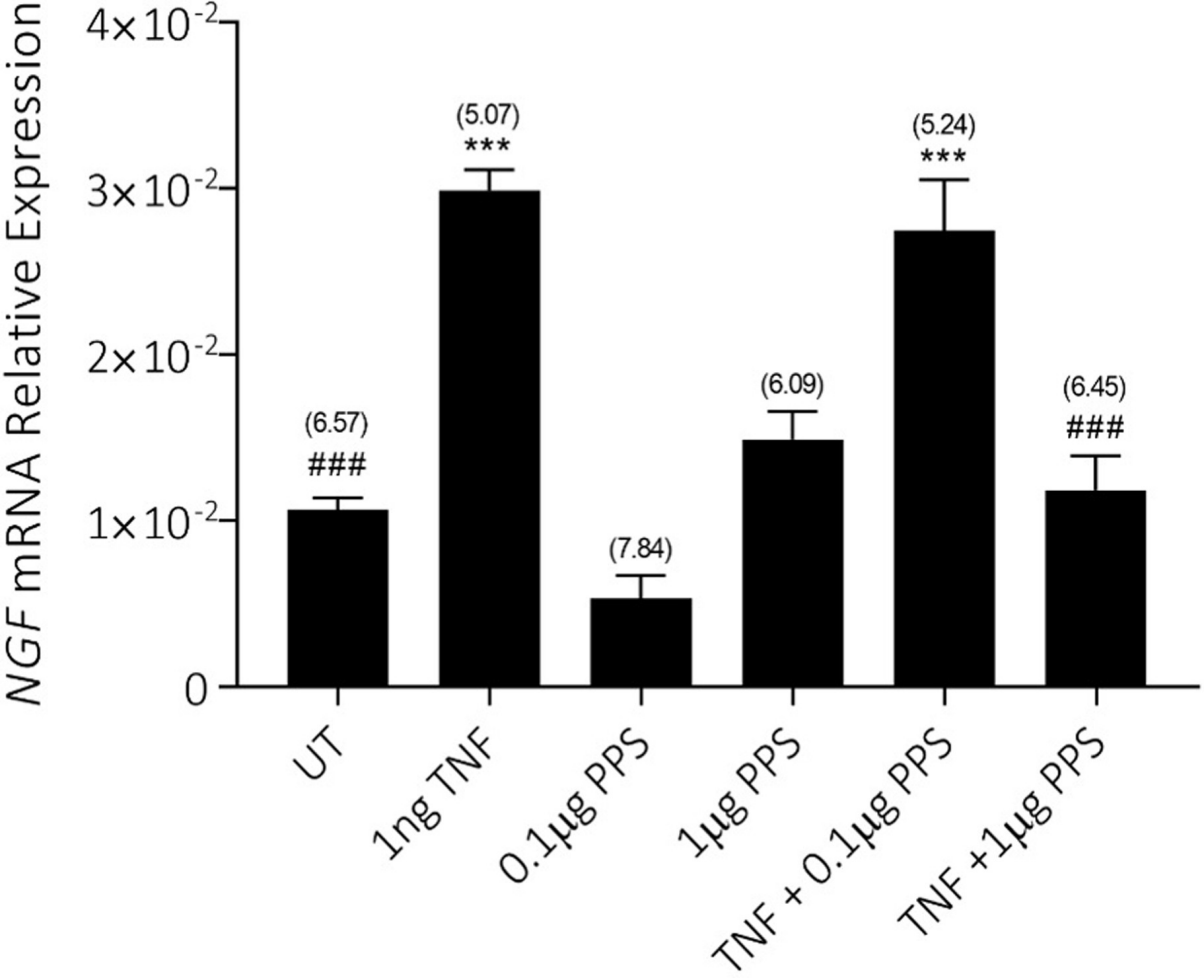
Effects of combinations of rhTNF and PPS on *NGF* gene expression in osteocyte-like cultures. Human differentiated osteocyte-like cultures were either untreated or pretreated with PPS (0.1 or 1.0 μg/ml) for 24h, then with or without rhTNFα (1 ng/ml) for a further 48h, and then real-time RT-PCR was performed for *NGF* mRNA. Data are mean + SD of triplicate real-time RT-PCR reactions normalised to the mRNA expression of the housekeeping gene *ACTB*.

To examine whether the effects of PPS on NGF/proNGF expression were at the transcriptional level, we also examined *NGF* mRNA expression using real-time RT-PCR (**Fig 5**). Exposure to TNFα increased the relative expression of *NGF* mRNA. PPS at both 0.1 and 1 μg/ml had no apparent effect on basal *NGF* mRNA levels. However, PPS at 1 μg/ml significantly reduced NGF expression in the presence of TNFα, suggesting that at least some of the effect of PPS on osteocytes is at the transcriptional level. This is consistent with a previous report that PPS acts as a transcriptional inhibitor of intracellular signalling pathways elicited by TNFα/TNF receptor signalling [13].

This finding is consistent with the protein expression data and supports the hypothesis that PPS reverses the effects of proinflammatory mediators in KOA on the expression of mediators of pain. The expression by osteocytes of two of the known receptors for NGF was also examined by RT-PCR as well as immunohistochemistry. The expression of the high affinity receptor tropomyosin receptor kinase A (TrkA) was not detectable by RT-PCR in any donor’s cells, consistent with a complete lack of signal by immunostaining/confocal microscopy (S3 Fig). The lack of expression of TrkA by human osteocytes is consistent with the findings of Castaneda-Corral *et al*., who reported that only neurons expressed detectable levels of this protein in mouse bone [33]. Very low levels of the NGF receptor *P75NTR* mRNA were however detected, although there was sporadic detection across the samples tested; as for TRKA, no detectable immunostaining for this molecule was observed (**S3 Fig**). These observations support the concept that NGF expression by osteocytes acts in a paracrine manner in the bone, with the most likely target cell being bone sensory neurons.

## Conclusions

This study shows for the first time the effects of PPS on human primary osteocytes isolated from the subchondral bone in patients with osteoarthritis of the knee. It is also the first demonstration of the production and secretion of NGF/proNGF by this cell type. PPS inhibited basal and TNFα-induced levels of proNGF secretion and TNFα induced *NGF* mRNA expression. PPS also inhibited TNFα-induced levels of the collagenase MMP-13. Together, this provides evidence that PPS may act at multiple levels to suppress the release of NGF and potentially other pain mediators in the subchondral bone, to ameliorate pain associated with knee osteoarthritis.

## Supporting information

S1 Fig Mineralising properties of KOA and NOF osteoblast/osteocyte-like cultures. Cultures of KOA or NOF cells were cultured under osteogenic differentiating conditions and stained at 3d, 14d and 28d for mineral deposition using the Alizarin Red technique, as described in Materials and Methods. Calcium deposition is indicated by red staining. Representative wells are shown for each donors’s cells at each time point.
(PPTX)

S2 Fig Human NOF osteocyte-like cells secrete proNGF. Secretion of proNGF was tested from cultures of NOF osteocyte-like cells treated with combinations of rhTNF and PPS. Data are means + SD of supernatants harvested from triplicate wells. Significant difference to untreated control (UT) is indicated by *(*p* < 0.05); significant difference to rhTNF treated cultures is indicated by #(*p* < 0.05).
(PPTX)

S3 Fig Immunostaining of KOA-derived osteocytes for TrkA and P-75. Day 28 differentiated human primary osteocyte-like cultures were immunostained and examined by confocal microscopy, as described in Materials and methods, for (A) TrkA (B) P-75 or were stained with an isotype control monoclonal antibody (C). Scale bars in each case represent 50 μm.
(PPTX)

## References

[1] Chevalier X, Eymard F, Richette P. Biologic agents in osteoarthritis: hopes and disappointments. Nat Rev Rheumatol. 2013;9(7):400–10. Epub 2013/04/03. 10.1038/nrrheum.2013.44 . 23545735

[2] Ayers DC, Li W, Harrold L, Allison J, Franklin PD. Preoperative pain and function profiles reflect consistent TKA patient selection among US surgeons. Clin Orthop Relat Res. 2015;473(1):76–81. Epub 2014/06/25. 10.1007/s11999-014-3716-5 24957788 PMC4390921

[3] Tomlinson RE, Li Z, Li Z, Minichiello L, Riddle RC, Venkatesan A, et al NGF-TrkA signaling in sensory nerves is required for skeletal adaptation to mechanical loads in mice. Proc Natl Acad Sci U S A. 2017;114(18):E3632–E41. Epub 2017/04/19. 10.1073/pnas.1701054114 28416686 PMC5422802

[4] Lane NE, Schnitzer TJ, Birbara CA, Mokhtarani M, Shelton DL, Smith MD, et al Tanezumab for the treatment of pain from osteoarthritis of the knee. N Engl J Med. 2010;363(16):1521–31. Epub 2010/10/15. 10.1056/NEJMoa0901510 . 20942668 PMC6896791

[5] Schnitzer TJ, Ekman EF, Spierings EL, Greenberg HS, Smith MD, Brown MT, et al Efficacy and safety of tanezumab monotherapy or combined with non-steroidal anti-inflammatory drugs in the treatment of knee or hip osteoarthritis pain. Ann Rheum Dis. 2015;74(6):1202–11. Epub 2014/03/15. 10.1136/annrheumdis-2013-204905 . 24625625

[6] Ekman EF, Gimbel JS, Bello AE, Smith MD, Keller DS, Annis KM, et al Efficacy and safety of intravenous tanezumab for the symptomatic treatment of osteoarthritis: 2 randomized controlled trials versus naproxen. J Rheumatol. 2014;41(11):2249–59. Epub 2014/10/03. 10.3899/jrheum.131294 . 25274899

[7] Takano S, Uchida K, Miyagi M, Inoue G, Fujimaki H, Aikawa J, et al Nerve Growth Factor Regulation by TNF-alpha and IL-1beta in Synovial Macrophages and Fibroblasts in Osteoarthritic Mice. J Immunol Res. 2016;2016:5706359 Epub 2016/09/17. 10.1155/2016/5706359 27635406 PMC5007361

[8] Bradshaw RA, Pundavela J, Biarc J, Chalkley RJ, Burlingame AL, Hondermarck H. NGF and ProNGF: Regulation of neuronal and neoplastic responses through receptor signaling. Adv Biol Regul. 2015;58:16–27. Epub 2014/12/11. 10.1016/j.jbior.2014.11.003 25491371 PMC4426037

[9] Malerba F, Paoletti F, Cattaneo A. NGF and proNGF Reciprocal Interference in Immunoassays: Open Questions, Criticalities, and Ways Forward. Front Mol Neurosci. 2016;9:63 Epub 2016/08/19. 10.3389/fnmol.2016.00063 27536217 PMC4971159

[10] Giusto LL, Zahner PM, Shoskes DA. An evaluation of the pharmacotherapy for interstitial cystitis. Expert Opin Pharmacother. 2018;19(10):1097–108. Epub 2018/07/05. 10.1080/14656566.2018.1491968 . 29972328

[11] Sampson MJ, Kabbani M, Krishnan R, Nganga M, Theodoulou A, Krishnan J. Improved clinical outcome measures of knee pain and function with concurrent resolution of subchondral Bone Marrow Edema Lesion and joint effusion in an osteoarthritic patient following Pentosan Polysulphate Sodium treatment: a case report. BMC Musculoskelet Disord. 2017;18(1):396 Epub 2017/09/14. 10.1186/s12891-017-1754-3 28899386 PMC5596862

[12] Kumagai K, Shirabe S, Miyata N, Murata M, Yamauchi A, Kataoka Y, et al. Sodium pentosan polysulfate resulted in cartilage improvement in knee osteoarthritis—an open clinical trial. (1472–6904 (Electronic)). 10.1186/1472-6904-10-7 PMC2873929 20346179

[13] Sunaga T, Oh N, Hosoya K, Takagi S, Okumura M. Inhibitory effects of pentosan polysulfate sodium on MAP-kinase pathway and NF-kappaB nuclear translocation in canine chondrocytes in vitro. The Journal of veterinary medical science / the Japanese Society of Veterinary Science. 2012;74(6):707–11. Epub 2012/01/05. 10.1292/jvms.11-0511 . 22214865

[14] Bwalya EC, Kim S, Fang J, Wijekoon HMS, Hosoya K, Okumura M. Pentosan polysulfate inhibits IL-1beta-induced iNOS, c-Jun and HIF-1alpha upregulation in canine articular chondrocytes. PLoS One. 2017;12(5):e0177144 Epub 2017/05/05. 10.1371/journal.pone.0177144 28472120 PMC5417682

[15] Atkins GJ, Findlay DM. Osteocyte regulation of bone mineral: a little give and take. Osteoporos Int. 2012;23(8):2067–79. Epub 2012/02/04. 10.1007/s00198-012-1915-z . 22302104

[16] Prideaux M, Findlay DM, Atkins GJ. Osteocytes: The master cells in bone remodelling. Curr Opin Pharmacol. 2016;28:24–30. 10.1016/j.coph.2016.02.003 . 26927500

[17] Ormsby RT, Cantley M, Kogawa M, Solomon LB, Haynes DR, Findlay DM, et al Evidence that osteocyte perilacunar remodelling contributes to polyethylene wear particle induced osteolysis. Acta Biomater. 2016;33:242–51. Epub 2016/01/23. 10.1016/j.actbio.2016.01.016 . 26796208

[18] Yang D, Wijenayaka AR, Solomon LB, Pederson SM, Findlay DM, Kidd SP, et al Novel Insights into Staphylococcus aureus Deep Bone Infections: the Involvement of Osteocytes. MBio. 2018;9(2). Epub 2018/04/25. 10.1128/mBio.00415-18 29691335 PMC5915738

[19] Atkins GJ, Rowe PS, Lim HP, Welldon KJ, Ormsby R, Wijenayaka AR, et al Sclerostin is a locally acting regulator of late-osteoblast/preosteocyte differentiation and regulates mineralization through a MEPE-ASARM-dependent mechanism. J Bone Miner Res. 2011;26(7):1425–36. Epub 2011/02/12. 10.1002/jbmr.345 21312267 PMC3358926

[20] Atkins GJ, Welldon KJ, Wijenayaka AR, Bonewald LF, Findlay DM. Vitamin K promotes mineralization, osteoblast-to-osteocyte transition, and an anticatabolic phenotype by {gamma}-carboxylation-dependent and -independent mechanisms. Am J Physiol Cell Physiol. 2009;297(6):C1358–67. Epub 2009/08/14. 10.1152/ajpcell.00216.2009 . 19675304

[21] Kogawa M, Wijenayaka AR, Ormsby RT, Thomas GP, Anderson PH, Bonewald LF, et al Sclerostin Regulates Release of Bone Mineral by Osteocytes by Induction of Carbonic Anhydrase 2. J Bone Miner Res. 2013;28(12):2436–48. Epub 2013/06/06. 10.1002/jbmr.2003 . 23737439

[22] Kumarasinghe DD, Sullivan T, Kuliwaba JS, Fazzalari NL, Atkins GJ. Evidence for the dysregulated expression of TWIST1, TGFβ1 and SMAD3 in differentiating osteoblasts from primary hip osteoarthritis patients. Osteoarthritis & Cartilage. 2012;20:1357–66. 22820497 10.1016/j.joca.2012.07.005

[23] Wijenayaka AR, Kogawa M, Lim HP, Bonewald LF, Findlay DM, Atkins GJ. Sclerostin stimulates osteocyte support of osteoclast activity by a RANKL-dependent pathway. PLoS One. 2011;6(10):e25900 10.1371/journal.pone.0025900 21991382 PMC3186800

[24] Ghosh P, Wu J, Shimmon S, Zannettino AC, Gronthos S, Itescu S. Pentosan polysulfate promotes proliferation and chondrogenic differentiation of adult human bone marrow-derived mesenchymal precursor cells. Arthritis Res Ther. 2010;12(1):R28 Epub 2010/02/20. 10.1186/ar2935 20167057 PMC2875662

[25] Prideaux M, Schutz C, Wijenayaka AR, Findlay DM, Campbell DG, Solomon LB, et al Isolation of osteocytes from human trabecular bone. Bone. 2016;88:64–72. Epub 2016/04/26. 10.1016/j.bone.2016.04.017 . 27109824

[26] Iannone F, De Bari C, Dell'Accio F, Covelli M, Patella V, Lo Bianco G, et al Increased expression of nerve growth factor (NGF) and high affinity NGF receptor (p140 TrkA) in human osteoarthritic chondrocytes. Rheumatology (Oxford). 2002;41(12):1413–8. Epub 2002/12/07. 10.1093/rheumatology/41.12.1413 . 12468822

[27] Zhang J, Wang LS, Ye SL, Luo P, Wang BL. Blockage of tropomyosin receptor kinase a (TrkA) enhances chemo-sensitivity in breast cancer cells and inhibits metastasis in vivo. Int J Clin Exp Med. 2015;8(1):634–41. Epub 2015/03/19. doi: Not available. 25785038 PMC4358493

[28] Rouillard AD, Gundersen GW, Fernandez NF, Wang Z, Monteiro CD, McDermott MG, et al. The harmonizome: a collection of processed datasets gathered to serve and mine knowledge about genes and proteins. LID—10.1093/database/baw100 [doi] LID—baw100 [pii]. (1758–0463 (Electronic)). 10.1093/database/baw100 PMC4930834 27374120

[29] Atkins GJ, Welldon KJ, Halbout P, Findlay DM. Strontium ranelate treatment of human primary osteoblasts promotes an osteocyte-like phenotype while eliciting an osteoprotegerin response. Osteoporos Int. 2009;20(4):653–64. 10.1007/s00198-008-0728-6 . 18763010

[30] Bonewald LF. The amazing osteocyte. J Bone Miner Res. 2011;26(2):229–38. Epub 2011/01/22. 10.1002/jbmr.320 . 21254230 PMC3179345

[31] Manni L, Aloe L. Role of IL-1 beta and TNF-alpha in the regulation of NGF in experimentally induced arthritis in mice. Rheumatol Int. 1998;18(3):97–102. Epub 1998/12/02. . 9833249 10.1007/s002960050065

[32] Dawes J Fau—Prowse CV, Prowse Cv Fau—Pepper DS, Pepper DS. Absorption of heparin, LMW heparin and SP54 after subcutaneous injection, assessed by competitive binding assay. Thrombosis Research. 1986;44(0049–3848 (Print)):683–93. 10.1016/0049-3848(86)90169-6 2433788

[33] Castaneda-Corral G, Jimenez-Andrade JM, Bloom AP, Taylor RN, Mantyh WG, Kaczmarska MJ, et al The majority of myelinated and unmyelinated sensory nerve fibers that innervate bone express the tropomyosin receptor kinase A. Neuroscience. 2011;178:196–207. Epub 2011/02/01. 10.1016/j.neuroscience.2011.01.039 21277945 PMC3078085

