# Supplementary material for: Human osteocyte expression of Nerve Growth Factor: The effect of Pentosan Polysulphate Sodium (PPS) and implications for pain associated with knee osteoarthritis
Source: PLoS One. 2019 Sep 26;14(9):e0222602. doi: 10.1371/journal.pone.0222602 (PMC6762051; doi:10.1371/journal.pone.0222602)
Supplement: S1 Fig — Cultures of KOA or NOF cells were cultured under osteogenic differentiating conditions and stained at 3d, 14d and 28d for mineral deposition using the Alizarin Red technique, as described in Materials and Methods. Calcium deposition is indicated by red staining. Representative wells are shown for each donors’s cells at each time point. (PPTX) [file pone.0222602.s001.pptx]

## Slide 1
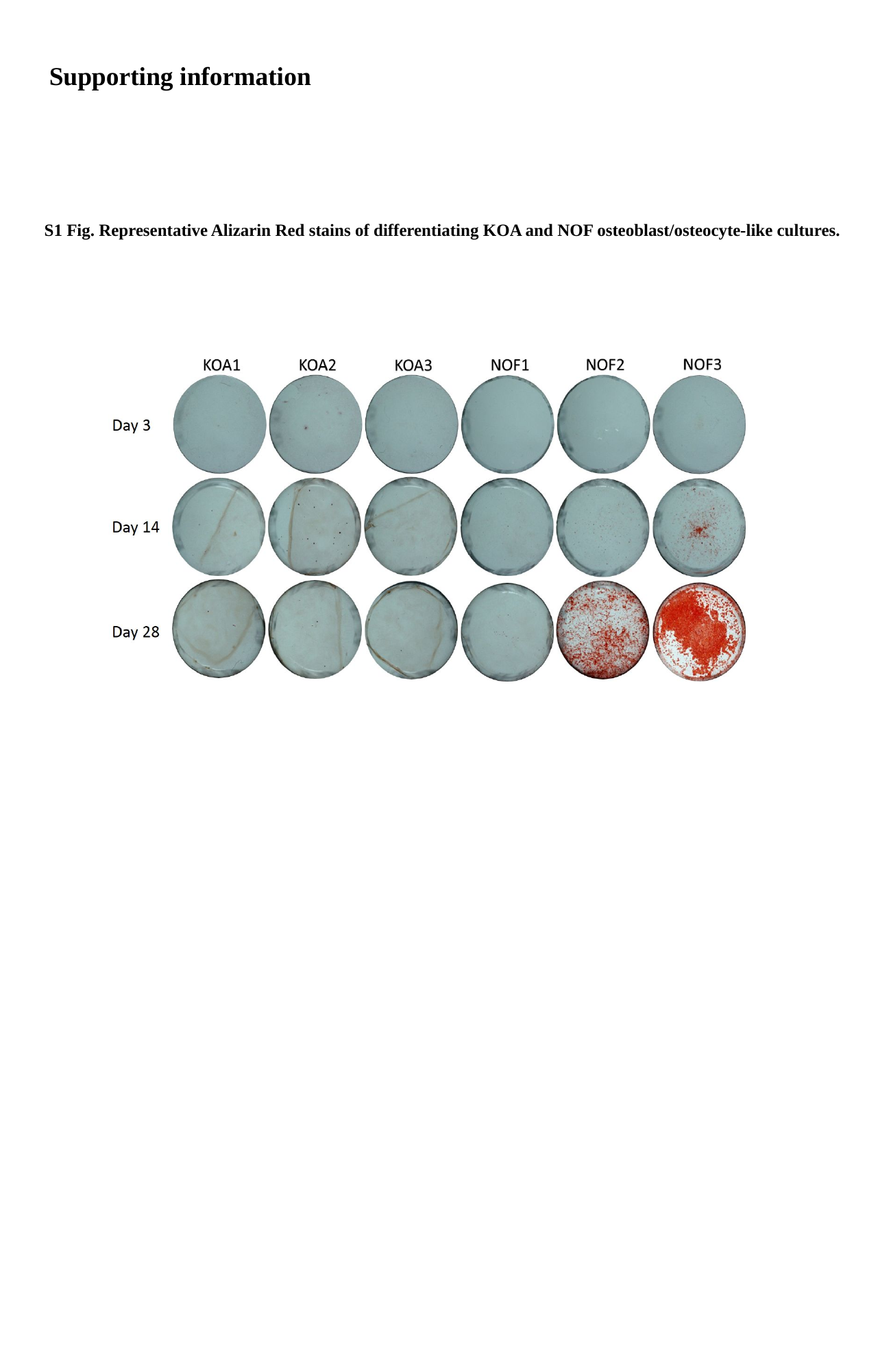

Supporting information
S1 Fig. Representative Alizarin Red stains of differentiating KOA and NOF osteoblast/osteocyte-like cultures.
