# Supplementary figures and images for: Human osteocyte expression of Nerve Growth Factor: The effect of Pentosan Polysulphate Sodium (PPS) and implications for pain associated with knee osteoarthritis
Source: PLoS One. 2019 Sep 26;14(9):e0222602. doi: 10.1371/journal.pone.0222602 (PMC6762051; doi:10.1371/journal.pone.0222602)

## Slide 1
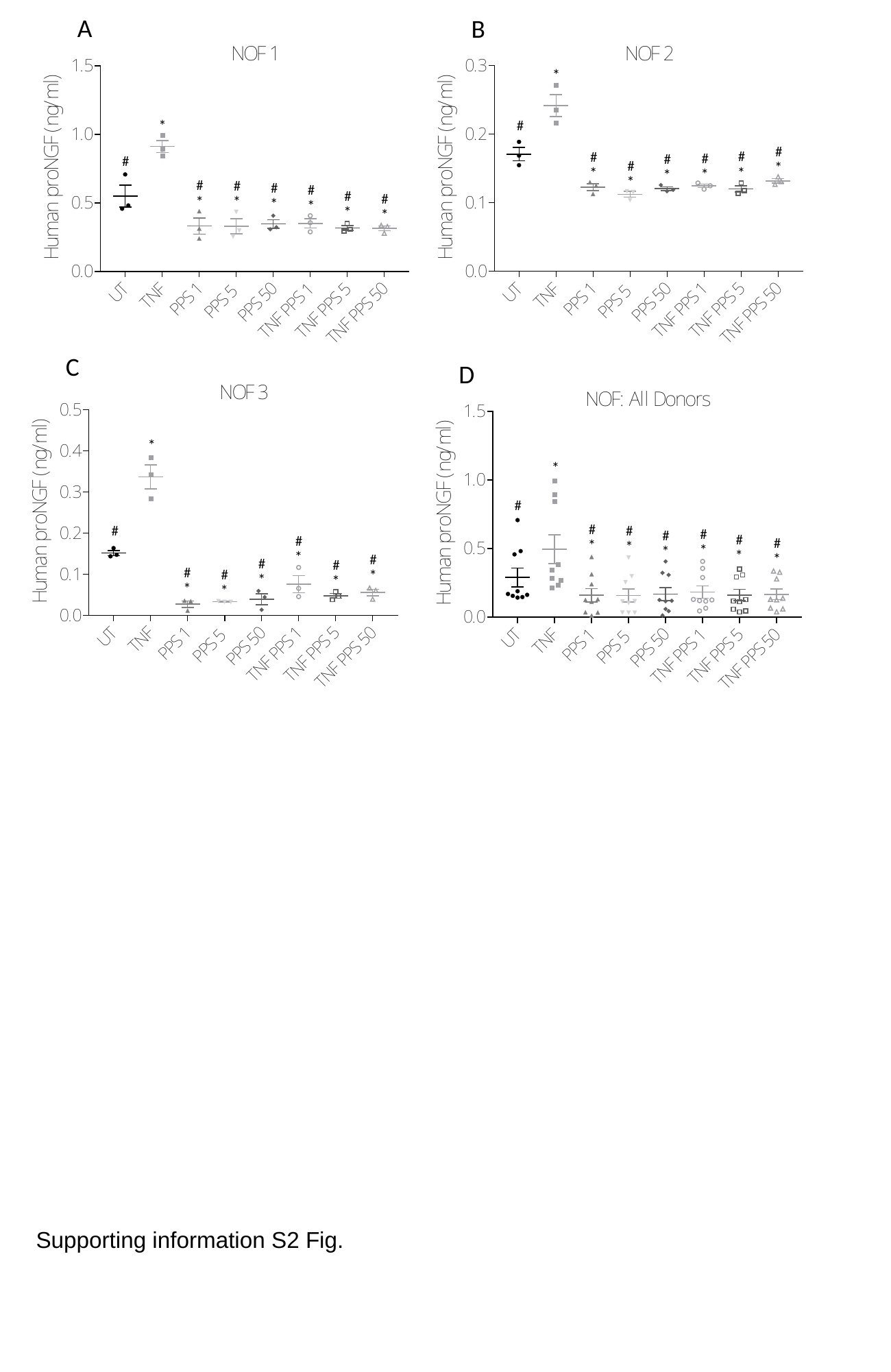

A
*
#
#
*
#
*
#
*
#
*
#
*
#
*
B
*
#
#
*
#
*
#
*
#
*
#
*
#
*
C
D
*
#
#
*
#
*
#
*
#
*
#
*
#
*
*
#
#
*
#
*
#
*
#
*
#
*
#
*
Supporting information S2 Fig.

Supplement: S2 Fig — Secretion of proNGF was tested from cultures of NOF osteocyte-like cells treated with combinations of rhTNF and PPS. Data are means + SD of supernatants harvested from triplicate wells. Significant difference to untreated control (UT) is indicated by *(p < 0.05); significant difference to rhTNF treated cultures is indicated by #(p < 0.05). (PPTX) [file pone.0222602.s002.pptx]

## Slide 1
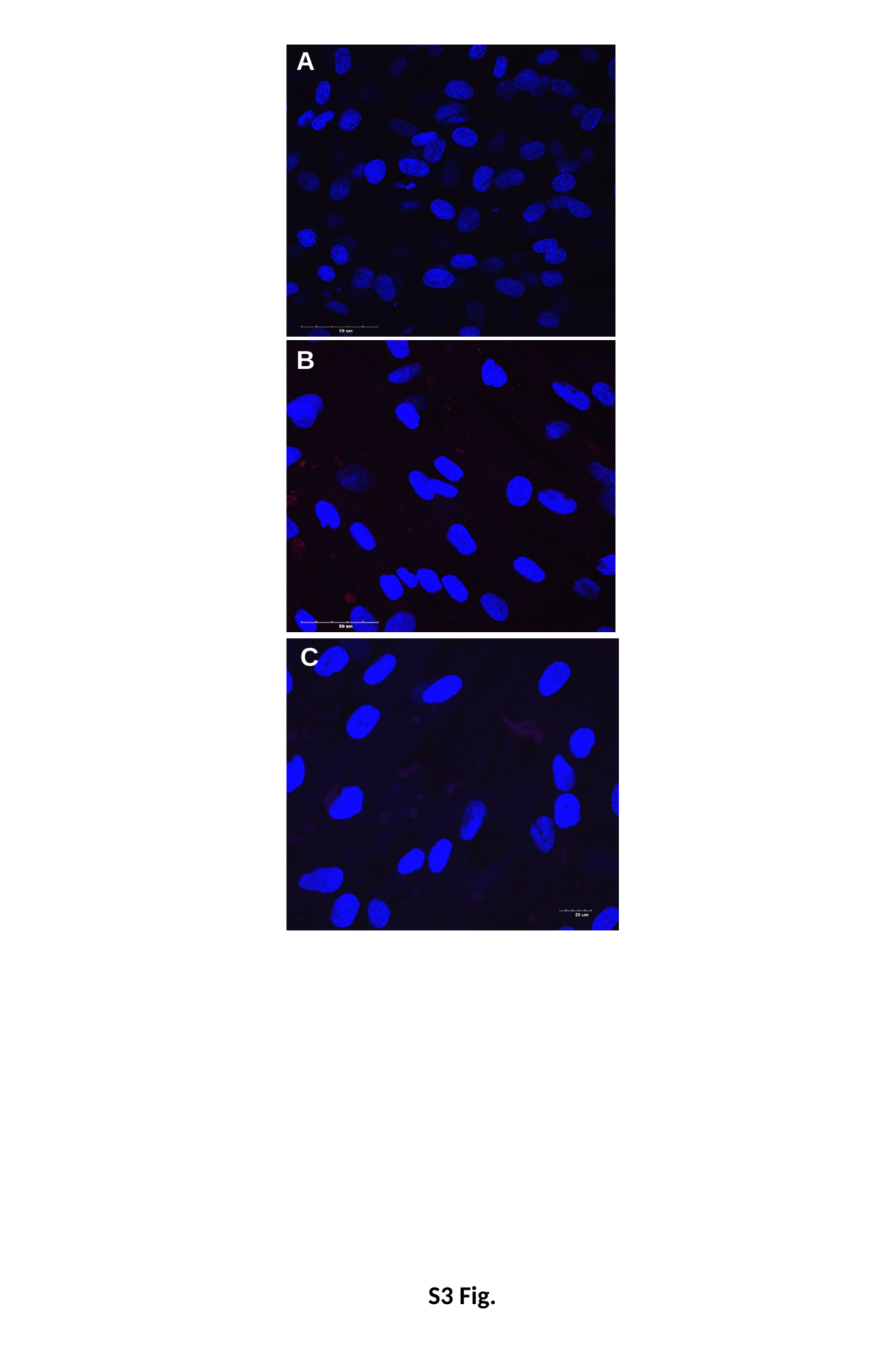

A
B
C
S3 Fig.

Supplement: S3 Fig — Day 28 differentiated human primary osteocyte-like cultures were immunostained and examined by confocal microscopy, as described in Materials and methods, for (A) TrkA (B) P-75 or were stained with an isotype control monoclonal antibody (C). Scale bars in each case represent 50 μm. (PPTX) [file pone.0222602.s003.pptx]
